# Origins of Oil and Gas Sector Methane Emissions: On-Site Investigations of Aerial Measured Sources

**DOI:** 10.1021/acs.est.2c07318

**Published:** 2023-01-30

**Authors:** Matthew R. Johnson, David R. Tyner, Bradley M. Conrad

**Affiliations:** Energy & Emissions Research Laboratory, Department of Mechanical and Aerospace Engineering, Carleton University, Ottawa, Ontario K1S 5B6, Canada

**Keywords:** methane venting, uncontrolled storage tanks, controlled storage tanks, compressors, combustion slip, unlit flares, occurrence rates, population emission factors

## Abstract

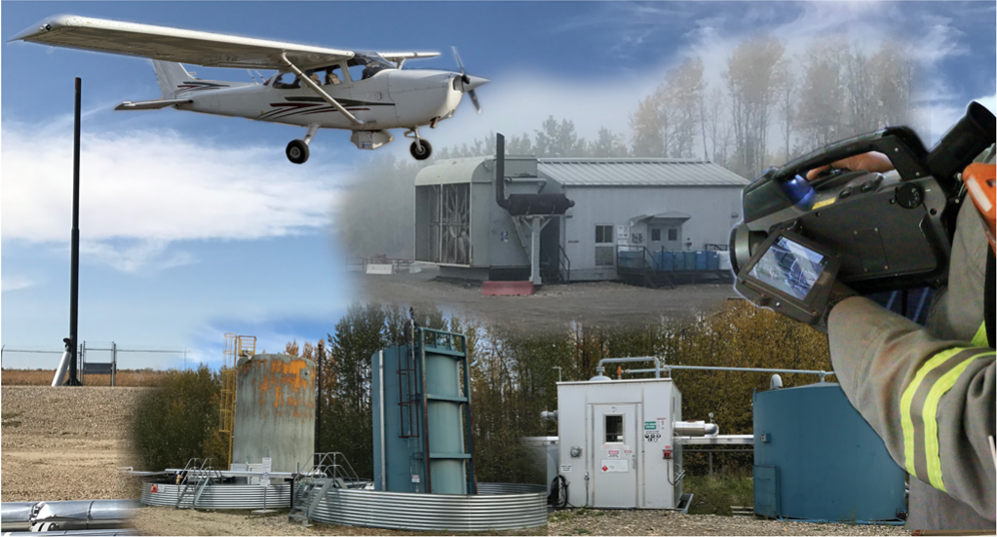

Success in reducing oil and gas sector methane emissions is contingent on understanding the sources driving emissions, associated options for mitigation, and the effectiveness of regulations in achieving intended outcomes. This study combines high-resolution, high-sensitivity aerial survey data with subsequent on-site investigations of detected sources to examine these points. Measurements were performed in British Columbia, Canada, an active oil- and gas-producing province with modern methane regulations featuring mandatory three times per year leak detection and repair (LDAR) surveys at most facilities. Derived emission factors enabled by source attribution show that significant methane emissions persist under this regulatory framework, dominated by (i) combustion slip (compressor exhaust and also catalytic heaters, which are not covered in current regulations), (ii) intentional venting (uncontrolled tanks, vent stacks or intentionally unlit flares, and uncontrolled compressors), and (iii) unintentional venting (controlled tanks, unintentionally unlit/blown out flares, and abnormally operating pneumatics). Although the detailed analysis shows mitigation options exist for all sources, the importance of combustion slip and the persistently large methane contributions from controlled tanks and unlit flares demonstrate the limits of current LDAR programs and the critical need for additional monitoring and verification if regulations are to have the intended impacts, and reduction targets of 75% and greater are to be met.

## Abstract 2

Origins, occurrence rates, and emission factors for key oil and gas methane sources reveal monitoring/reporting/verification and regulatory gaps that need attention to reach crucial methane emissions targets.

## Introduction

Near-term, aggressive mitigation of methane emissions in the oil and gas sector is a central part of international efforts to temper climate change. More than 100 countries have recently committed to the Global Methane Pledge^1^ intended to achieve 30% reductions in methane emissions by 2030. Most of these reductions will come in the oil and gas sector, which is the dominant source of methane emissions in many countries and has the greatest potential for near-term mitigation. Indeed, the International Energy Agency has called for a 75% reduction in oil and gas sector methane emissions by 2030 as a required step toward a 2050 target for net-zero emissions and an essential part of keeping a <1.5 °C temperature rise within reach;^2−4^ Canada has recently committed to this target as part of its Global Methane Pledge.^5,6^ In parallel, the European Commission has announced an intent to regulate “compulsory monitoring, reporting, and verification” (MRV) of oil and gas methane emissions, specifically obligating these requirements for all imported fossil energy into the European Union.^7^

The ability to monitor and verifiably reduce methane is contingent on understanding the sources driving emissions and associated options for mitigation. Numerous recent studies have consistently demonstrated higher-than-reported or -inventoried methane emissions throughout the upstream oil and gas sector via a range of aerial,^8,9^ ground sensor-based,^10^ vehicle-based,^11−13^ and satellite^14^ measurement approaches. However, because emissions can be driven by a diversity of potential sources, understanding the specifics of individual sources and the origins of observed detections is essential for developing effective regulations and driving mitigation. Although recent work in British Columbia, Canada has provided direct insight into major emitting equipment—identifying storage tanks, compressors, and unlit flares as dominant contributors to methane emissions from upstream production sites^9^—there remains a dearth of data on source-specific drivers of emissions and emission rates.

This paper directly addresses this gap through a detailed analysis of source-resolved, high-sensitivity aerial methane measurements combined with on-site investigations of detected emitters. Measurements were completed in British Columbia (BC), Canada, an active oil- and gas-producing region that produces ∼36% of Canada’s natural gas^15^ and has the potential to become an important global gas exporter with the completion of the LNG Canada liquified natural gas terminal currently under construction.^16^ More importantly, as noted in previous studies, upstream production sites in BC are typical of conventional oil and gas operations throughout North America and many parts of the world, with a diversity of facilities and major sources that include compressors, storage tanks, flares, separators, and other common equipment.^9^ Finally, BC is also an example of a jurisdiction with modern methane regulations, which since January 2020 has featured requirements for three times per year leak detection and repair (LDAR) surveys at most facilities.^17^ In this sense, sources examined in this study are examples of what persists under a prescribed LDAR program and the further challenges that remain in eliminating methane emissions as part of reaching net-zero emission objectives. Implications are thus expected to be especially relevant to any other jurisdiction currently implementing or considering regulated LDAR as part of their methane mitigation efforts.

## Methods

Aerial measurements were completed using Bridger Photonics’ Gas Mapping LiDAR (GML)^18,19^ at 508 upstream oil and gas production sites in BC, Canada during September–October 2021 (see Figure S1 in the Supporting Information, SI). As part of a parallel effort to develop measurement-based methane inventories,^20^ these 508 sites (representing 59.5% of active facilities and 8.1% of active wells) were chosen to be broadly representative of upstream oil and gas facilities in the province and included a range of active well sites, single- and multiwell oil and gas facilities, compressor stations, gathering facilities, and gas plants. Based on detailed models derived from controlled release data,^21^ for typical flight altitudes in the present survey (mean and 95% reference range of 176 and 145–218 m above ground level) the GML technology enables detection and quantification of sources as small as 0.6–4.2 kg/h (at 50% probability of detection) depending on conditions (including wind speed, which was obtained from Meteoblue (meteoblue.com) in the present survey) with typical single- and multiple-pass measurement uncertainties of −69/+113 and −46/+54%, respectively, at 95% confidence.

For each site in the survey, an initial flight was completed that entailed 1–13 partially overlapping passes to fully cover the facility considering the ∼100 m wide laser measurement swath. For sites with detected emissions, a second flight (“reflight”) was performed 1–11 days later to re-screen detected sources and re-quantify emissions. As in Tyner and Johnson,^9^ data from individual passes (ranging from 1 to 9 passes per detected source with a median of 3 and mean of 3.1) were averaged^20^ by flight, and results for each flight were averaged to compute measured emission rates for each unique detected source location. Robust emission rate uncertainties were calculated via Monte Carlo analysis using the uncertainty models detailed in Conrad et al.^21^ Outputs from the GML included geolocated plumes at ∼1 m resolution overlaid with high-resolution aerial imagery. Following the methods detailed by Tyner and Johnson,^9^ these aerial data were combined with facility plot plans and production accounting information (both obtained by request from the BC Oil and Gas Commission, BC OGC); commercially available pipeline and infrastructure data (Geomatics Data Management, Inc.); and any additional collected information from on-site inspections (see below) to identify individual methane sources and associated facilities.

Additionally, as soon as possible (within 1–15 days; median and mean of 6 and 7.7 days, respectively) following the initial flights, optical gas imaging (OGI) technicians subcontracted from Greenpath Energy Ltd. were deployed to investigate detected sources to glean information about the origins of detected emissions. Working in close collaboration with the BC Government, the ground team was able to access facilities accompanied by a BC OGC inspector. The primary objective of these ground inspections was to investigate specific sources associated with initial aerial detections and where possible refine source attribution or suggest contributing sources. In addition to collecting OGI videos of detected plumes and photos of emitting equipment, while on site, the ground team was tasked with creating an inventory of active compressors, including the make/model and type of the driving engine and whether the vented emissions were controlled. For each uncontrolled compressor, whether it was being investigated to determine specific drivers of aerial detected sources or not, the technicians performed a quick OGI scan of associated vent lines to screen for detectable venting. Similarly, an inventory of all controlled and uncontrolled liquid storage tanks was completed while on site. For uncontrolled tanks, when visibly accessible, roof-top vents were also quickly screened for detectable venting via OGI. Although budget constraints precluded deploying the ground team to all sites, they were able to complete follow-up inspections at 75 of 184 facilities with aerial measured emissions. These sites were chosen primarily based on availability of real-time data from the concurrent aerial survey flights, prioritizing closely located sites and sites with more sources if there were multiple sites to choose from. In total, the ground team investigated 195 of 543 aerial detected sources. Collected OGI imagery, photos, and field notes were sufficient to confirm or attribute likely sources for 192 of these, including 81 sources attributed to combustion slip as further discussed below. For two other cases, there was confusion on the site orientation such that potential sources could not be definitively matched to aerial sources. The final source (a plume associated with a group separator as further noted below) was not seen by the ground crew but wind conditions were noted to be very strong (>8 m/s) during their visit. This implies that the 1–15 day delay between flights and ground visits was not important relative to the apparent persistence of detected sources.

## Results and Discussion

**Figure 1:**
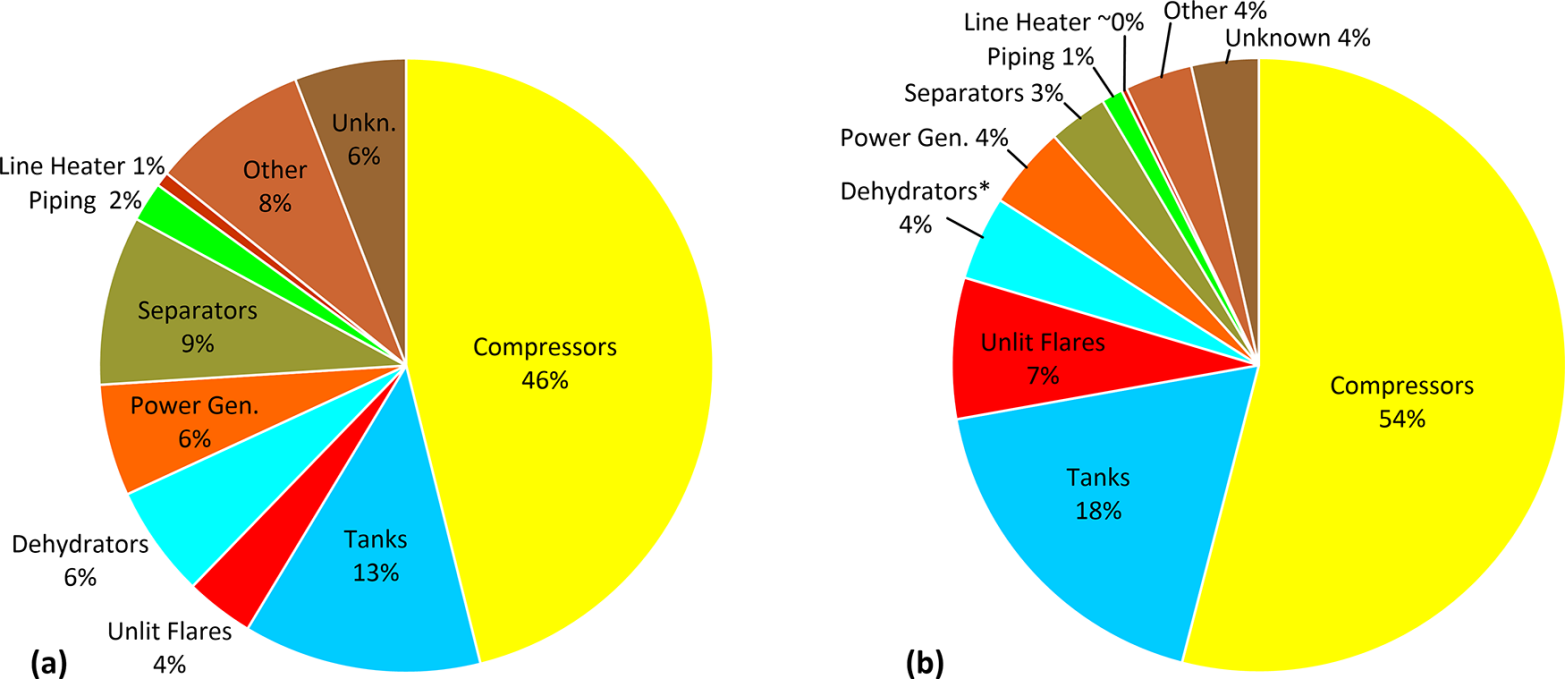
Relative contribution of source types to aerially detected methane. (a) Fraction of detected sources and (b) fraction of measured methane among quantified source types in the aerial survey. *Dehydrator emissions are commonly routed through associated tanks.

Within the sample of 508 upstream oil and gas production sites surveyed by the plane, there were 527 quantifiable sources from 184 distinct sites. An additional 16 sources were detected but could not be quantified, which included sources at four additional sites. Compressors and tanks are the two most frequently detected source categories (Figure 1a) as well as the two largest contributors to measured methane emissions (Figure 1b). Although unlit and poorly performing flares are less commonly detected than separators, dehydrators, power generation equipment, or other/unknown sources, they can be individually large sources and thus have a disproportionate impact as the third largest methane contributor in the quantified source breakdown. Together, compressors, tanks, and unlit flares represent 80% of aerial measured methane emissions at upstream oil and gas sites in BC; considering that most dehydrator emissions are also routed through associated tanks, this fraction grows to 84%.

This measured source breakdown is similar to that seen in a smaller 2019 survey of 167 sites in BC^9^ with a few key differences. While the same three key sources (compressors, tanks, and unlit flares) dominate emissions, compressors are much more important and unlit flares and tanks less important in the present data. There are several likely reasons for this difference. First, the present study is much more comprehensive with more than three times the number of aerially surveyed sites and corresponding ground follow-ups implemented in quick succession, which together enable a much better-resolved source breakdown. Second, significant regulatory changes were implemented in the intervening year (2020) including a three times per year LDAR requirement. Third, it is likely that provincial, national, and international attention on unlit flares following recent publications^9,22,23^ and incoming (January 2023) limits on tank venting in BC^17^ have incited early action by producers to mitigate these critical sources. Conversely, compressor engine emissions are not captured in current regulations as further discussed below. Detailed analysis of each major source type is discussed in the sections that follow.

### Compressors

Compressors, or more precisely the “compressor package” consisting of a natural gas engine or electric motor coupled with a compressor, can be especially challenging sources to quantify and mitigate as their emissions can include uncombusted methane in the engine exhaust (commonly known as combustion/methane slip); intentionally vented methane from compressor rod packing, crankcase, or lubricating oil vents; and/or unintentional emissions from failing emission control systems or leaks. Moreover, compressors are most commonly housed in buildings that may include additional methane sources such as natural gas-driven pneumatic equipment, regulators, catalytic heaters (commonly known as “Cata-Dyne heaters”), or other equipment, piping, fittings, etc. While OGI cameras are useful in detecting noncombustion sources of methane, they are unable to detect methane within combustion plumes such as engine exhaust. This gap is important since extractive sampling field measurements have demonstrated the importance of this combustion engine methane slip as a major source of methane emissions at compressor stations.^24^

OGI technicians were deployed to investigate aerial detected methane sources attributed to 93 different compressor buildings within 75 sites. In addition, while on this same set of sites, they inventoried 192 active reciprocating compressors and documented (when possible) the make and model of the driving system and whether the compressor had controls in place to capture and redirect vented emissions (i.e., rod packing vents, crankcase vents, and lube oil vents). When present, these control systems included directing vented sources to flare systems, into on-site vapor recovery units, or into the compressor suction. For compressors without controls, the ground team also used OGI cameras to quickly screen whether there were detectable emissions from any of the vent lines associated with the compressor. Note that because combustion slip could not be directly observed using OGI cameras on the ground, it was necessarily identified via the process of elimination (i.e., if no other OGI-detectable sources associated with the compressor building were observed, the aerially detected source was deemed to be associated with combustion slip) backed by manual review of the high-resolution plume imagery that identified sources clearly originating from the compressor exhaust stacks.

**Figure 2:**
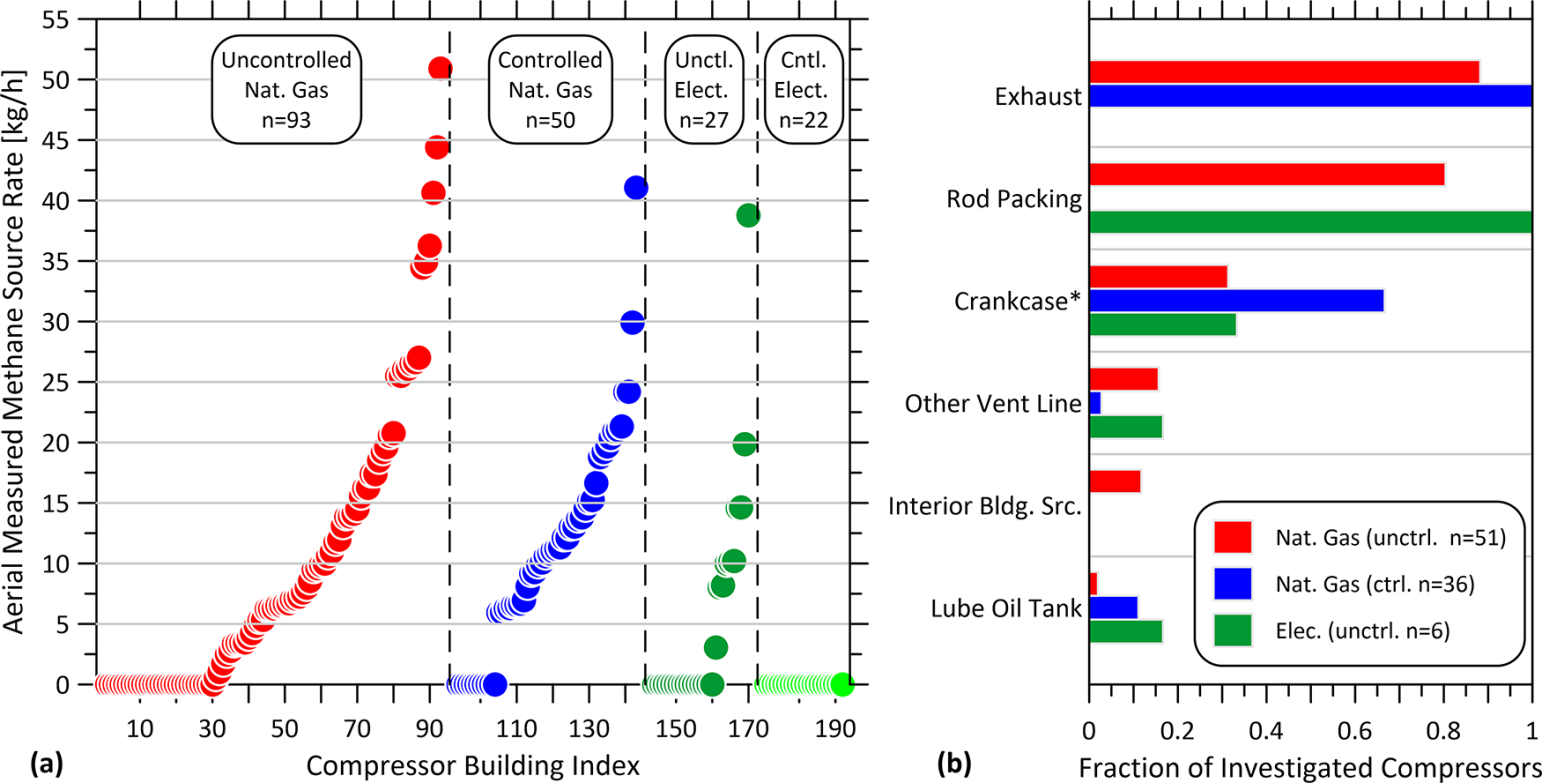
Aerial measured methane emissions from compressors. (a) Magnitudes of methane emissions associated with 192 reciprocating compressors; (b) suggested contributing origins of observed emissions from ground investigations of 93 emitting compressors. Crankcase* includes 8 uncontrolled and 23 controlled natural gas-fired compressors where the crankcase vent was tied into the engine exhaust.

Figure 2a shows the aerial measured source rates for different categories of controlled and uncontrolled compressor packages. Despite no explicit regulation toward electrification, 25% (49/192) of compressor packages in the field sample were found to be electric drive. For the subset of these with controlled vent sources, none had detected emissions in the aerial survey (0/22), which shows that zero-emission solutions are technically and economically feasible (when electricity is available) and already used in practice. Investigations of aerial detected sources associated with compressor packages (Figure 2b) attributed emissions primarily to methane slip in the combustion exhaust (for natural gas drive compressors) and rod packing vents for uncontrolled gas- and electric-drive units. For controlled natural gas drive compressors, more than 60% of investigated units also had detectable venting emissions from the crankcase suggesting these additional sources may be commonly overlooked. The relative contributions of these sources can be gleaned from detection rate and population emission factor data by compressor configuration and engine type, derived by combining aerial quantifications with collected on-site unit-specific data as summarized in Table 1.

**Table 1:** Comparison of Detection Frequency of Compressor Sources by Ground-Based OGI and Aerial Measurements and Derived Population Emission Factors from Aerial Data

compressor package type	detection frequency of vented sources by OGI ground crew^a^	detection frequency by aerial GML^a^	population emission factor based on GML measured sources (95% confidence limits)
gas fired—uncontrolled	87% (76/87)	68% (63/93)	9.6 kg/h (8.7–10.8)
gas fired—controlled	b	78% (39/50)	11.1 kg/h (10.0–12.4)
electric drive—uncontrolled	96% (26/27)	37% (10/27)	5.1 kg/h (3.9–6.8)
electric drive—controlled	b	0% (0/22)	0.0 kg/h

a Numbers in parentheses (*n*/*N*) specify the number of compressors with detected emissions (*n*) and the total number of surveyed compressors (*N*).

b Controlled compressors were only screened by the ground team when there was an associated aerial detected source to investigate.

Counterintuitively, the population-averaged measured methane emission rate for controlled natural gas engine-driven compressors was slightly higher than uncontrolled compressors, although the 95% confidence intervals (derived from Monte Carlo analysis using GML uncertainty models from Conrad et al.^21^) overlap. This suggests that aerial detected emissions are dominated by methane slip in the engine combustion exhaust consistent with the results of Zimmerle et al.^25^ who found that “combustion slip is the largest category of methane emissions at gathering stations” in the U.S. The dominant contribution of combustion slip is also reflected in the similar (slightly higher) aerial detection rates for controlled vs uncontrolled natural gas drive compressors and the much lower average emissions measured for uncontrolled electric drive units. The ground team qualitatively observed vented emissions from 96% (26/27) of uncontrolled electric drive compressors, whereas only 37% (10/27) of these units were detected from the air, suggesting that noncombustion compressor package emissions were captured in the aerial measurements ∼40% of the time.

**Figure 3:**
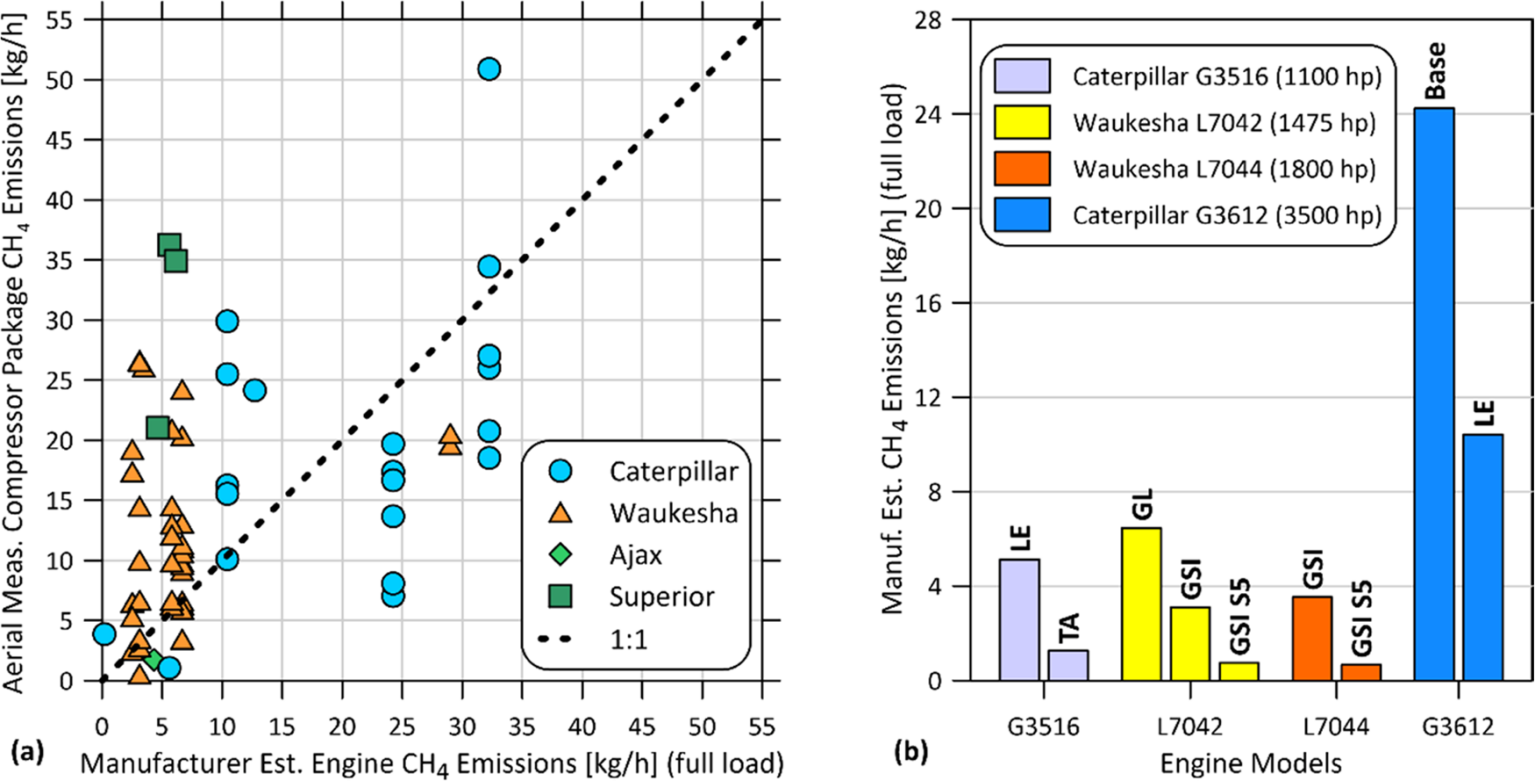
Measured and manufacturer-specified compressor methane emissions. (a) Comparison of aerial measured compressor package/compressor building methane emissions (total emissions) with expected methane emissions in combustion engine exhaust from manufacturer specifications. (b) Examples of variation in manufacturer-rated methane emissions for different compressor engine models within a series. Labels on bars indicate manufacturers’ model identifiers from spec sheets—Caterpillar: base model; LE = low emissions (lean burn); TA = (rich burn); Waukesha: GL = (lean burn), GSI (rich burn), and GSI S5 (rich burn with series 5 emissions control system).

To investigate this further, Figure 3a compares aerial measured total compressor package-related emissions to expected manufacturer-rated methane emissions for the specific makes and models of compressor engines inventoried by the ground team. Notwithstanding the presence of aftermarket fuel-air controllers on some units and the unknown operating loads at the time of aerial measurement, the measured emissions vs expected combustion slip emissions from the associated engines fall over the 1:1 line in the figure. Measured emissions above the correspondence line reflect a combination of elevated combustion slip relative to manufacturer-rated performance plus added emissions from noncombustion and nonengine sources. The general correspondence confirms the dominance of methane slip in overall compressor-related emissions.

Apart from electrification—i.e., replacing natural gas-fired engines with electric motors as found at one-quarter of installations—there are important policy questions about what other options exist to reduce methane slip in compressor exhaust. Figure 3b explores this question comparing manufacturer-rated methane emissions for different models of compressors of a given size class (brake horsepower; bhp). For example, Waukesha, the manufacturer accounting for the largest share (59%) of the 135 identifiable natural gas-driven compressor engines in the survey, reports 5–9 times lower methane emissions for their Series 5 (S5) rich burn engines versus their lean burn GL and GSI models. This difference between rich and lean burn engines is consistent with differences seen in the field measurements of Vaughn et al.^24^ Similar potential methane emissions differences are seen in the plotted example from Caterpillar, who represent 36% of gas-driven compressor engines in the survey data. A closer analysis of the present sample of controlled compressors found that only lean burn units were detected in the aerial survey, whereas detected uncontrolled compressors included a mix of lean and rich burn units. However, ignoring control status, aerial measured emissions (including zeroes) from identifiable rich burn and lean burn compressor engines were equivalent on a size-adjusted per brake horsepower basis. Apart from the small sample size and potential confounding contributions of vented sources, one possible reason for higher-than-expected emissions in the field sample of rich burn engines is the prevalence of aftermarket fuel-air control systems. Among all compressors, those noted to have fuel-air control systems had higher average measured methane emissions, both on an absolute basis and on a per brake horsepower basis. It is possible that some past efforts using retrofitted systems for rich-to-lean engine conversions as a means of controlling NO_*x*_ emissions without the need for a catalytic converter have exacerbated methane emissions. By contrast, the more modern engine control systems incorporating three-way catalytic conversion can simultaneously reduce NO*_x_* and hydrocarbons.^26^ Existing manufacturer programs for retrofittable compressor upgrades (e.g., Waukesha reUp^27^), generally marketed on the basis of reduced operating and maintenance costs, may also have notable benefits for methane reduction in cases where full electrification is not feasible.

### Storage Tanks

Venting through liquid storage tanks is often cited as a primary source of methane emissions throughout the upstream oil and gas sector.^9,28−31^ Indeed, recent analysis by Rutherford et al.^32^ found storage tank emissions were the largest source category in their revised U.S. oil and gas sector inventory and the single largest contributor to discrepancies with official estimates. However, they also noted that tanks are especially poorly characterized, with available tank data used in their modeling limited to a single campaign in Fort Worth, Texas.^33^

Within the 508 sites in the aerial survey, 79 emitting tanks were detected and quantified from 61 distinct sites not counting emissions from an additional five tanks that were detected but could not be quantified. This site-level tank emission detection rate of 12% is similar to the 11% rate seen in a 2015 qualitative helicopter-based OGI survey of well pads in North Dakota,^31^ which detected emissions only at sites with on-site tanks. For the subset of sites visited by the ground crew, field notes and collected count data were used to definitively identify active tanks and whether controls were present. For the remaining sites, high-resolution aerial imagery and facility plans (when available) were used to manually count the total number of tanks flown during the survey, noting that some inactive tanks are likely included in this estimate such that subsequently derived population emission factors are expected to be conservatively low. To the extent possible, these same data were used to classify each tank (i.e., production tank, dehydrator/condensing tank, compressor lube oil tank, amine tank) as well as ascertain the presence of emission controls (see the SI).

**Figure 4:**
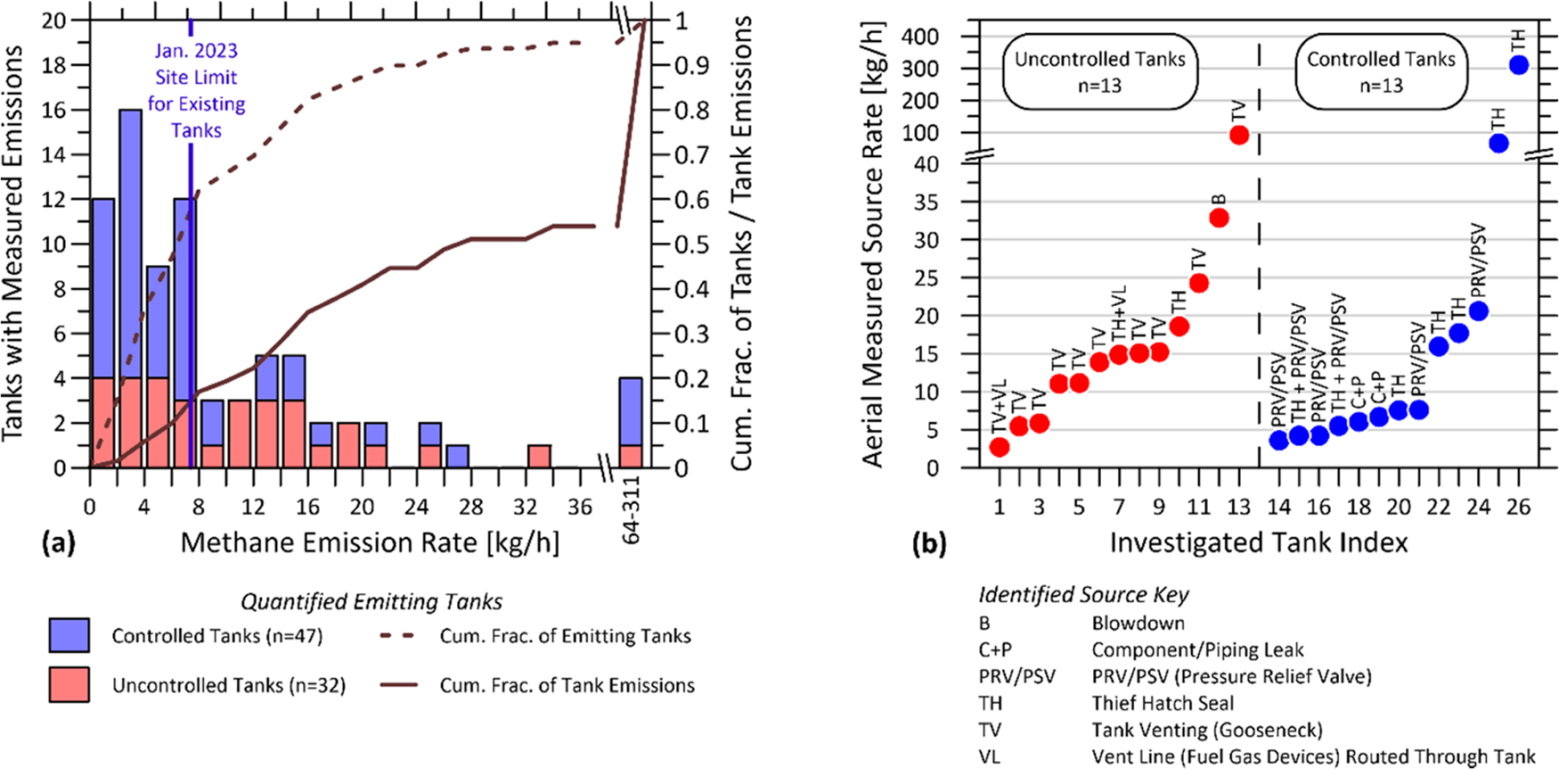
Analysis of the origins of methane emissions from controlled and uncontrolled liquid storage tanks. (a) Distribution of emission rates from 79 detected and quantified tanks in the aerial survey. (b) Identified sources from on-site investigations of emitting tanks.

As is typical in the oil and gas sector, Figure 4a shows that the distribution of tank sources is strongly skewed to the right. Curiously, examples of strongly emitting controlled tanks and uncontrolled tanks are both found over the range of the distribution, with the largest source in the sample (311 kg/h) coming from a controlled tank with an open thief hatch. This is especially surprising given that BC currently has regulations in place requiring three times per year LDAR inspections that should theoretically be capturing and fixing/reducing emission events from controlled tanks.^17^Figure 4b shows the identified origins for the subset of aerial detected tanks investigated by the ground crew. Emissions at uncontrolled tanks were all attributed to forms of direct venting, whether through the gooseneck tank vent or thief hatch. In two investigated cases, fuel gas-driven devices were also being vented through the uncontrolled tank. Investigated sources at controlled tanks included pressure relief valves, thief hatch seals, and leaks of associated components and piping. This is consistent with attributions by Lyman et al.^29^ who found that controlled tanks most commonly emitted “not from the control devices themselves, but from tank hatches, vents, or piping upstream of the control devices”.

As part of creating a count inventory of on-site tanks and controls, the ground technicians were also tasked with using handheld OGI cameras to screen for emissions from all uncontrolled tanks where there was visual access to the gooseneck vent. Noting that the technicians were preferentially dispatched to sites with sources (although not necessarily tank sources) as part of identifying origins and/or causes of emissions, they detected emissions from 57% (44 of 77) of uncontrolled tanks vs 18% (23 of 127) for the aerial survey at this same subset of sites. This difference highlights that not all sources are captured in aerial measurements and is consistent with different lower detection limits of OGI (approximately 0.4 kg/h or better in favorable conditions when professionally deployed^34^) and the aerial GML (∼0.6–4.2 kg/h depending on wind speed and aircraft altitude^21^). However, considering the skewed distribution of measured tank emissions (Figure 4a), undetected tank sources below the aerial GML sensitivity should represent a negligibly small contribution to total measured emissions from tanks. Across all sites in the survey, the aerial GML detected emissions at 6.8% of identified uncontrolled tanks as detailed in Table 2.

**Table 2:** Detection Rates and Derived Emission Factors for Uncontrolled, Controlled, and All (Combined) Tanks Included in the Aerial Survey

tank control status	aerial detection rate^a^	emitter average from aerial detections (95% confidence limits) [kg/h/emitting tank]	population emission factor from aerial detections (95% confidence limits) [kg/h/tank]^a^
Production Tanks			
uncontrolled	6.0% (26^b^/430)	14.9 (12.1, 19.2)	0.80 (0.65, 1.03)
controlled	4.0% (38^c^/949)	16.8 (12.1, 24.9)	0.64 (0.46, 0.95)
combined	4.6% (64^b^,^c^/1379)	16.1 (12.9, 21.2)	0.69 (0.55, 0.91)
All Tanks (Incl. Tanks at Dehydrators, Condensing Tanks, Amine Tanks, Common Lube Oil Tanks)			
uncontrolled	6.8% (35^b^/512)	12.8 (10.7, 15.9)	0.80 (0.67, 1.00)
controlled	4.6% (49^c^/1069)	15.9 (12.1, 22.2)	0.70 (0.53, 0.98)
combined	5.3% (84^b^,^c^/1581)	14.6 (12.1, 18.5)	0.73 (0.61, 0.93)

a The total count and control status of tanks in the aerial survey (i.e., tanks at sites where the ground crew was not dispatched) were estimated from aerial imagery and facility plans. This total number of tanks is likely conservatively high as it could include inactive tanks, leading to conservatively low detection rates and population emission factors for active tanks.

b Includes three uncontrolled tanks that were detected as emitting but not quantified.

c Includes two controlled tanks that were detected as emitting but not quantified.

Combining on-site count data and aerial quantifications, it is possible to derive both emitter average and population average emission factors for uncontrolled, controlled, and all (combined) tanks (Table 2). Somewhat surprisingly, the population emission factors for controlled tanks are only marginally lower than those for uncontrolled tanks and the 95% confidence limits significantly overlap. However, this is also consistent with qualitative survey results of Lyman et al.^29^ who found that “pads with tank emission controls were more likely to have detected emission plumes” suggesting that “malfunctioning tank emission control systems are very common”. What is surprising is that the average emissions for controlled tanks are so high given existing regulated three times per year LDAR programs as noted above. One explanation could be that tanks with controls tend to handle larger volumes of gas than tanks without controls such that a control failure has the potential to lead to much larger emissions. Indeed, closer inspection of the data reveals that the average controlled tank emissions are strongly influenced by the largest 311 kg/h tank with a failed thief hatch. As a thought experiment, recalculating the population emission factor without this site would reduce average controlled tank emissions from 0.70 to 0.41 kg/h. However, what this shows is that controlled tanks require proper design and sizing as well as continued monitoring to ensure reductions are achieved. Indeed, this same issue was sufficiently concerning that the U.S. Environmental Protection Agency (EPA) issued a compliance alert on this topic^35^ “to help operators assess whether their vapor control systems are properly designed, sized, operated, and maintained”.

### Unlit Flares

Across all sites in the survey, 360 potential flares were identified from high-resolution aerial imagery and facility plans. Of these, 188 (52%) were visually confirmed to have a lit flame without detected emissions, 19 (5.3%) had detected emissions with or without a flame as further detailed in Table S1 in the SI, and the remaining 153 (42.5%) had no visible flame and were either not emitting and inactive, or emitting below detection limits. Further investigation of the 19 sources revealed eleven unlit flares, three unlit vent stacks (“cold flares”), three partially lit flares (observed to be unlit and emitting during one flight but lit without detectable methane during a second flight), and two poorly performing lit flares with consistent methane emissions over two separate flights. Although collectively these represented only ∼4% of detected sources in the aerial survey (sixth most common source), they were the third largest contributor to aerial measured methane emissions (Figure 1). This is because individual unlit flares tend to be large sources, with the largest in the sample exceeding 100 kg/h of methane, averaged over two separate days. Notably, the 4% fraction of detected sources is lower than the 10% source fraction seen in a sample of 167 sites in 2019.^9^ While it is possible this lower rate is due to the advanced warning of aerial surveys given in 2021 as part of arranging logistics for follow-up ground inspections, this does not diminish the achieved reduction and highlights the potential ease of addressing this source and the value of third-party MRV as a critical tool to drive mitigation.

Surprisingly, on-site investigations by the ground team revealed that at least two of the unlit flares were intentional, including the third largest source in Table S1 (50.1 kg/h at BC_367) which was being intentionally operated as a continuous vent. This is in addition to the three sites with measured emissions from unlit “vent stacks” as identified on facility plans. A common anecdotal argument for venting over flaring is that the emitted methane is insufficient to justify continuous operation of a flare, which may produce additional CO_2_ from pilot and purge flows. However, this is seldom, if ever, true in practice. On a continuous basis, any vented methane source larger than 0.089 kg/h produces greater equivalent greenhouse gas (GHG) emissions than a 4-inch plain-end flare at maximum recommended purge and pilot flow rates (see SI Section S2). Moreover, this threshold reduces to zero as purge and pilot requirements are reduced (e.g., using electronic ignition and/or baffle seals) or if a combustor system without continuous pilot/purge requirements is used to destroy methane.

For intermittent vent sources, the potential trade-off in GHG emissions between venting and flaring depends on the duration and frequency of venting relative to the operating time of the flare system in the event the latter is kept running continuously on standby. To investigate this further as elaborated in the SI, the final column of Table S1 calculates the maximum duration at the measured vent rate in hours, above which GHG emissions would be reduced even if maintaining an operating flare on continuous standby for a month and assuming no further vent releases occur in that month. For comparison, Table S1 also shows the time interval between the initial flight and subsequent reflight of each source, and whether the flare was unlit and/or detected in both flights. For the 11 unlit flares, 9 were emitting detectable methane during both flights 1–10 days apart, which was 9–180× longer than the duration to justify continuous operation of a flare. The other two unlit flares were confirmed to be unlit in both flights of the aerial surveys but were either not emitting or emitting below detection limits on the second flight. Nevertheless, at the measured emission rates for these sites, venting of any longer than 1–18 hours would produce more GHG emissions than flaring with continuous pilot and purge for an entire month. Moreover, the approved facility plans for all eleven sites suggest these flares should not be unlit as confirmed by BC OGC. Similarly, for the three facilities operating with approved vent stacks, the measured emission rates were more than sufficient to warrant GHG reductions via combustion. Although the smallest site was only surveyed once, the larger two sources (9.6 and 27.9 kg/h) were detected on both flights multiple days apart suggesting nontrivial vent volumes both from a GHG perspective and in the context of provincial regulations that specify comparatively lower limits for total venting from on-site tanks.^17^ This suggests an important gap in current regulations.

A further three flares were partially lit during the surveys, i.e., unlit and emitting during one flight but lit and not emitting detectable methane in the other flight. Although the specific reasons for these flares being unlit is unknown, more generally the propensity for flares and pilots to extinguish in high wind conditions is commonly acknowledged (e.g., refs (36, 37)). Although existing regulations in BC require “an adequate auto-ignition system” for “unsupervised flare stacks”,^17^ the continued prevalence of unlit flares highlights their contribution to methane emissions and the importance of continued monitoring. Methane emissions were also measured from two flares that were visibly lit but still emitting detectable methane during two separate flights. Notwithstanding that one of these was a pit flare which is no longer recommended for use,^38^ this implies issues with flare performance. These issues are not at all restricted to BC; a prominent recent survey in the U.S. Permian basin using helicopter-based infrared optical gas imaging found 5% of surveyed flares were unlit and a further 6% had “apparent combustion issues”.^22^ Although research is ongoing to develop better models to predict flare efficiency and methane slip over a range of conditions,^39^ in the absence of such models, this again highlights the need for regular MRV.

### Separators and Other Sources

**Figure 5:**
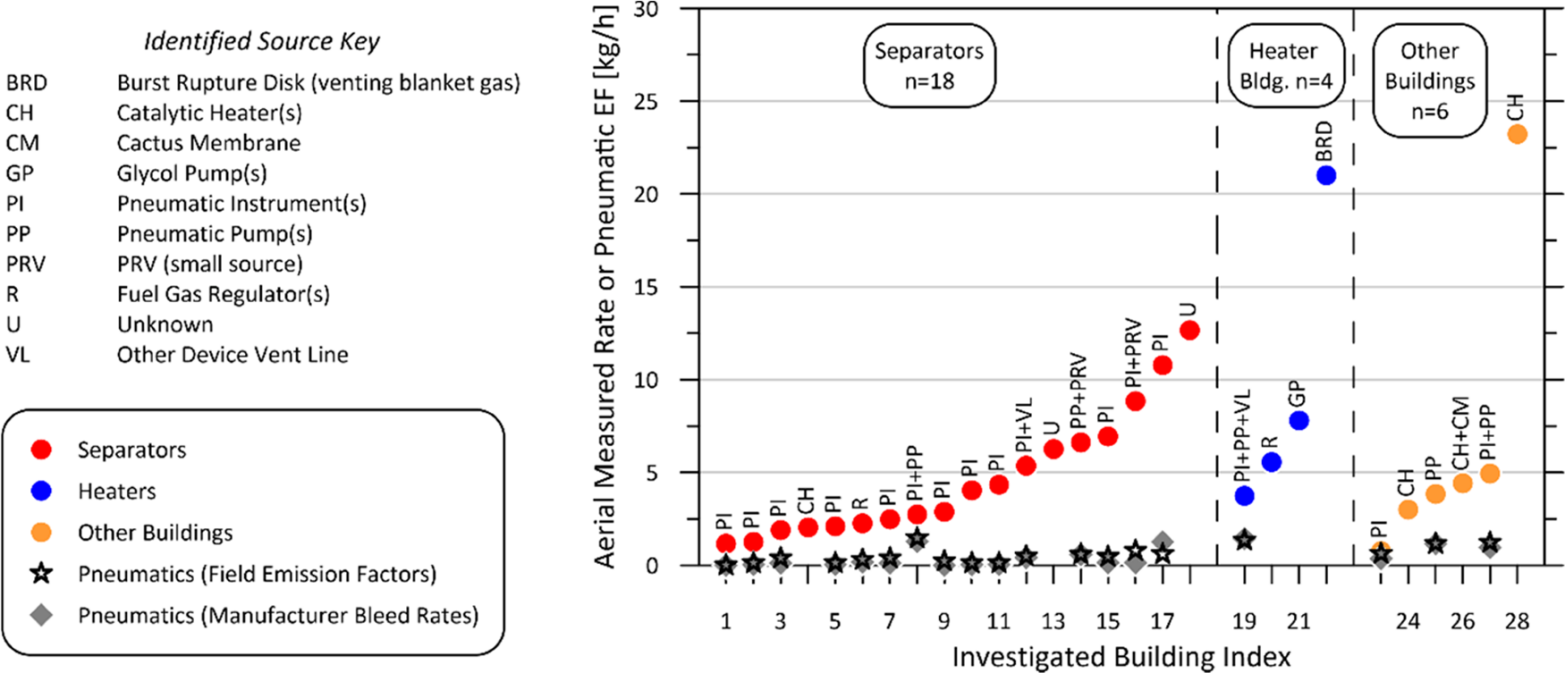
Investigated sources of aerial measured methane emissions at separators, heater buildings, and other buildings (i.e., three pump buildings, a manifold building, a fuel gas skid, and a gas lift building). Corresponding stars and diamonds indicate maximum expected emissions from on-site pneumatic equipment and pumps based on either summarized emission factors from field studies^40^ or manufacturer-specified bleed rates, respectively, for the specific makes/models of pneumatic equipment present at each site.

Remaining source types identified in the aerial survey included separators, heater buildings, and other buildings. The ground technicians were dispatched to investigate sources at 28 of these as summarized in Figure 5. Using OGI cameras, methane emissions were observed or likely sources were implicated such as natural gas-driven pneumatic devices and catalytic heaters (which, because any slipped methane is emitted with combustion products, are not observable via OGI). From the data shown in the figure, the six largest emitters were attributed (in decreasing order) to catalytic heaters, a burst rupture disk from a tank leading to venting of blanket gas, unknown (identified as a persistent source associated with a group separator vent measured in two flights 5 days apart but not seen by the ground technician presumably due to noted high winds and poor background sky conditions), pneumatic instruments and a pressure relief valve, and a glycol pump. Although the ground crew was not equipped to measure sources, in cases where pneumatic equipment and pumps were found, they were able to record specific makes and models of each pneumatic device. This enabled calculation of the expected methane emissions for the specific on-site pneumatic equipment based on manufacturer/model-specific data (see the SI). The gray and black symbols in Figure 5 show these expected methane emissions calculated using either manufacturer-specific bleed rates or emission factors from field studies as summarized in Manual 015 of the Alberta Energy Regulator.^40^ These results show that, although pneumatic equipment was commonly identified as a potential contributing source at these sites, they were either not the primary source or could only have been operating abnormally. Again, given that these sites are currently subject to three times per year LDAR with required repair schedules, this demonstrates the importance of independent MRV to overcome the limits of LDAR programs in practice and ensure regulations are being followed.

### Implications

Detailed analysis of the preceding aerial and ground survey data shows that in a jurisdiction with existing regulated three times per year LDAR requirements, methane emissions are dominated by (i) combustion slip from compressor exhaust and potentially catalytic heaters, (ii) intentional venting from uncontrolled tanks, vent stacks or intentionally unlit flares, uncontrolled compressors, and to a much lesser degree pneumatic equipment, and (iii) unintentional venting from controlled tanks and unlit/blown out flares and abnormally operating pneumatics. Ongoing efforts to regulate and reduce methane emissions will need to focus on these key sources. Moreover, a consistent theme throughout the preceding analysis is the need for independent MRV to detect and identify significant sources that persist under current LDAR programs. For compressor package emissions, the largest source category in the survey, data suggest that emissions are dominated by methane slip in the exhaust which cannot readily be seen by OGI cameras and is not currently regulated. However, the strong penetration of electric drive compressors coupled with the analysis of rich vs. lean burn compressors consistent with the detailed work of Vaughn et al.^24^ suggests that there is significant and practical mitigation potential from these sources using existing approaches. In addition, analysis of specific compressor engine models and controls hints that operational decisions to reduce NO*_x_* emissions from existing engines may have a role in exacerbating methane emissions. Based on these results, it is recommended that future regulations incentivize electrification of compressor drivers as much as possible, particularly for new installations given that compressor packages may have a field life of thirty years or more. Additionally, specific performance standards or limits on allowable methane emissions are recommended given the apparent significance of compressor engine exhaust as a source and the potential to unintentionally exacerbate methane emissions in an effort to reduce NO*_x_*. Further, the inability of OGI cameras to detect methane slip in compressor engine exhaust (or in the exhaust of other combustion sources such as catalytic heaters) as part of standard LDAR surveys highlights the need for complementary MRV approaches to screen and identify high-emitting compressor engines/other combustion-related sources and associated mitigation opportunities.

Although the vast majority of tanks in the survey did not emit detectable methane from the air, those that did emit were significant. Most surprising however was the apparent contribution of controlled tanks to total tank emissions, which was unexpected given that current LDAR regulations should be capturing these sources. This apparent gap has a direct impact on the anticipated effectiveness of current regulations in BC or in any jurisdiction implementing or considering similar LDAR requirements. Figure S2 in the SI aggregates tank emissions by site to compare with new (Jan. 1, 2023) regulatory limits for total site emissions from existing tanks.^17^ Half (52%) of sites in the 2021 survey had aggregate measured tank emissions above this new limit of 9000 m^3^/mo (whole gas, equivalent to 7.36 kg/h of methane), and these sites represent 90% of the total measured methane from tanks. In principle, bringing these sites into compliance with the limit could reduce emissions by 70%. Unfortunately, a closer analysis of the data suggests this is not realistic given that approx. 65% of these excess tank emissions are from tanks that already have controls and are currently subject to regulated 3 times per year LDAR surveys. Ideally, if regulations were working as intended, these emissions should already be reduced toward zero. Moreover, if the current measured population average for controlled tanks is taken as the achievable limit without additional MRV, then the upcoming tank limit is only likely to achieve a reduction of 27%. Similarly, unlit or partially lit flares and intentional vents remain as the third most important source of measured emissions but should also be one of the easiest to mitigate sources.

While it is possible that even more frequent LDAR surveys could speed the detection and mitigation of emitting tanks and flares, this is not a guaranteed solution given field data suggesting OGI-based LDAR can miss key sources in practice.^9^ In particular, visual access to tank tops and flare stacks can be challenging within the effective range of an OGI camera and would be compounded by factors such as reduced detection sensitivity at low temperatures and/or low temperature difference between plume and background.^34,41,42^ Moreover, LDAR reporting data in BC suggest regulatory compliance is also an important factor, both in terms of the fraction of facilities completing all required surveys and delays in the repair of detected sources.^43^ On this latter point, at least two emitting controlled tanks and one unlit flare from the present survey were also detected two years earlier as part of a much smaller survey.^9^ Finally, a closer analysis of available LDAR reporting data in BC shows significantly lower magnitudes of methane sources found in internal versus third-party LDAR surveys.^43^ This may be at least partially attributable to differences in training and experience, consistent with controlled field trials suggesting “surveyor experience and affiliation had the largest impact on detection rate”.^34^ All of these factors highlight the need for separate, independent monitoring and verification as an essential complement to standard LDAR programs. Ultimately, reaching near-term 75% reduction targets and subsequent net-zero goals will require combined efforts to mitigate all major methane sources (i.e., combustion slip, intentional venting, and unintentional venting) identified and investigated in this study.

## Supporting Information Available

Map of survey sites; discussion and analysis of GHG emissions trade-off between flaring and venting; and tank emissions aggregated by site (PDF)
Site-anonymized, aerially measured, Monte Carlo-averaged emissions for all attributed sources; site-anonymized inventory of compressors; site-anonymized inventory of tanks; and site-anonymized inventory of pneumatics linked with separators and other sources (XLSX)
