# Supplementary material for: Origins of Oil and Gas Sector Methane Emissions: On-Site Investigations of Aerial Measured Sources
Source: Environ Sci Technol. 2023 Jan 30;57(6):2484–94. doi: 10.1021/acs.est.2c07318 (PMC9933527; doi:10.1021/acs.est.2c07318)
Supplement: Supplementary file 1 — es2c07318_si_001.pdf [file es2c07318_si_001.pdf]

Supporting Information

Origins of Oil and Gas Sector Methane Emissions: On-Site Investigations of Aerial Measured Sources

Matthew R. Johnson*, David R. Tyner, Bradley M. Conrad

Energy & Emissions Research Laboratory,
Department of Mechanical and Aerospace Engineering,
Carleton University, Ottawa, ON, Canada, K1S 5B6

*Corresponding author: matthew.johnson@carleton.ca; 613-520-2600 x4039

File contains: 2 figures and 6 tables

S1 Study Region S2
S2 Greenhouse Gas Emissions of Flaring Versus Venting S2
S3 Total Site Tank Emissions S5
S4 References S6
S5 Underlying Data Tables S7

S1 Study Region

Figure S1 shows the locations of the aerial survey sites within the province of British Columbia, Canada and identifies sites with detected emissions (via airborne gas mapping LiDAR) and those investigated during follow-up visits by ground technicians using optical gas imaging cameras. These aerial survey sites are also considered in parallel work developing and demonstrating a protocol to produce measurement-based methane inventories[1].

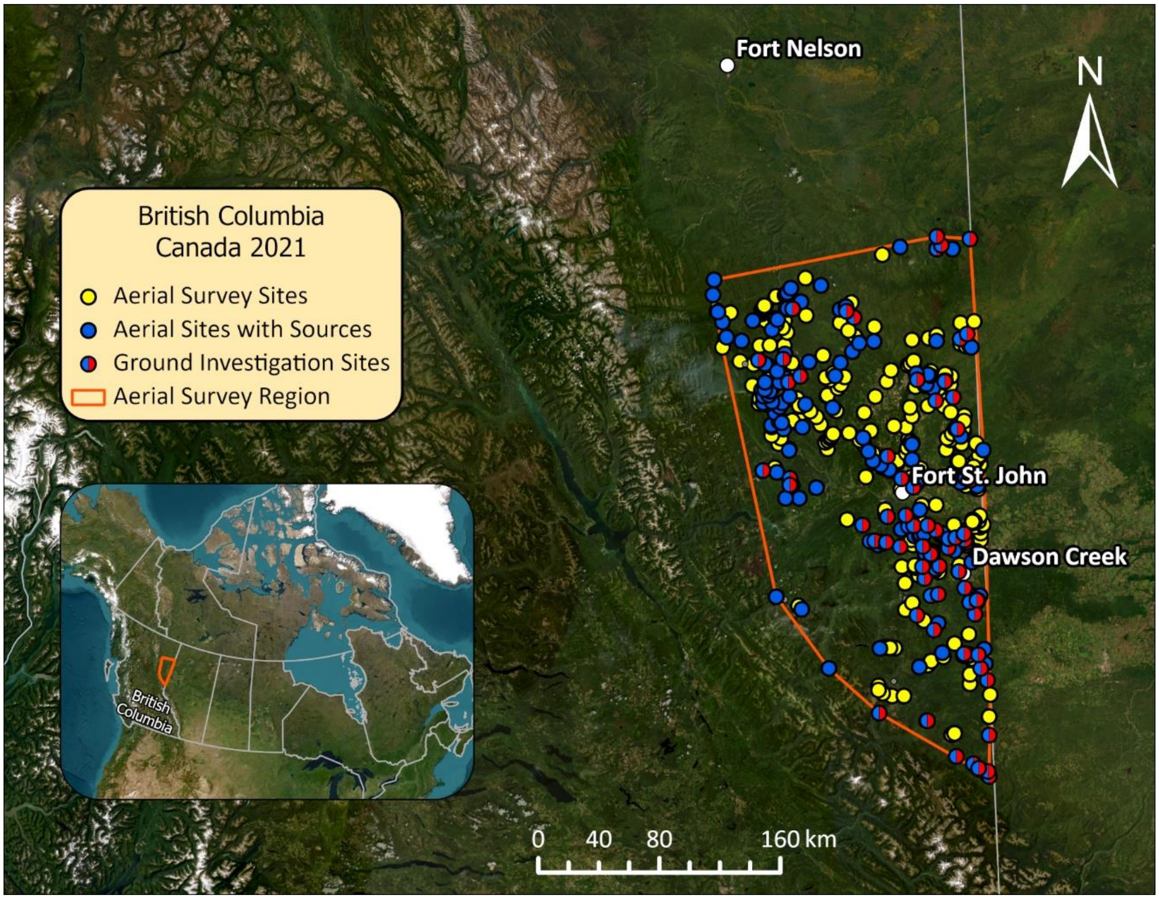

Figure S1. Map of present (2021) aerial survey region showing sites in the aerial survey and the subsets with detected emissions and ground investigation.

S2 Greenhouse Gas Emissions of Flaring Versus Venting

Because the radiative forcing of fossil methane is approximately 82.5/29.8 times greater than carbon dioxide on a mass-basis when evaluated on a 20/100 year time horizon[2], combustion of methane to carbon dioxide

will always reduce equivalent greenhouse gas (GHG) emissions relative to venting. However, for industrial flare systems where additional fuel may be required for pilot and purge systems to keep the flare operating and available, it is prudent to consider the potential added GHG emissions of these flows. Notably, continuous flare systems generally do not require purge gas[3]; it is possible to significantly reduce or eliminate purge flow requirements on intermittent flares through the use of seals[3–5] or flame arrestors[6]; and/or associated GHG emissions can be avoided through the use of nitrogen purge systems[4,5]. Similarly pilot flames may not be required for flares using electronic ignition systems. Nevertheless, in the most conservative case, maintaining an operating flare system to handle intermittent gas releases would incur GHG emissions from both purge and pilot flows. These GHG emissions can be calculated assuming recommended pilot[4] and purge[5] flow rates as summarized in the "Fuel Gas Best Management Practices" guide from Canadian Association of Petroleum Producers (CAPP)[3].

The recommended flow rate for a natural gas pilot in the CAPP guide is 1.98 m^3/h (which is sourced from the 70 scf/hr value specified in the EPA Air Pollution Control Cost Manual[4]). For a standard 4-inch diameter flare considering the worst case of a plain end flare with no seals (maximum flow condition), the recommended[3,5] purge flow is 0.34 m^3/h. Assuming an average gas composition from Table 6 in Tyner and Johnson[7] (i.e., 91.87% CH_4, 2.84% C_2H_6, 1.27% C_3H_8, 0.62% C_4H_{10}, 0.22% C_5H_{12}, 0.08% C_6H_{14}, 0.09% C_7H_{16}, 0.73% CO_2, plus other non-carbon, non-GHG species), GWP_{CH4}=82.5 (as relevant for near-term 2025, 2030, and 2050 targets), and conservatively assuming that both the pilot and purge gas burn with 98% carbon conversion efficiency, then continuous operation of pilot and purge would produce 6.91 kg_{CO2e}/h. Consider then a vented gas source of 104.09 m^3/month (whole gas) with the same composition such that it includes 0.089 kg_{CH4}/h of methane. Flaring this gas at 98% carbon conversion efficiency would produce 0.424 kg_{CO2e}/h (calculated assuming the emissions are driven by stripping of unburned fuel[8,9]). Conservatively assuming the flare still requires the full 6.91 kg_{CO2e}/h for pilot and purge, this equates to total GHG emissions of 7.33 kg_{CO2e}/h. Direct venting of this gas would produce the same 7.33 kg_{CO2e}/h. Thus, allowing for pilot and purge flow, flaring will reduce GHG emissions of any vented gas stream greater than 104.09 m^3/month (whole gas) or methane source greater than 0.089 kg_{CH4}/h. Repeating these calculations assuming GWP_{CH4}=29.8 suggests flaring will reduce GHG emissions of any vented gas stream greater than

241.6 m^3/month (whole gas) or methane source greater than 0.206 kg_{CH4}/h. A similar calculation is included in CSA Standard Z620.3:22[10].

As shown in Table S1 below, for each measured unlit flare or intentional vent stack, it is possible to calculate the maximum duration of that vent beyond which net greenhouse gas emissions would be lower via flaring even if it meant operating a 4-inch diameter flare continuously on standby for a ***month*** at recommended maximum pilot and purge flow rates. This is done by iteratively calculating the vent duration such that the vented gas GHG emissions match the GHG emissions incurred from flaring that same volume of gas plus the GHG emissions from maintaining an operating 4-inch diameter flare at maximum recommended purge and pilot flow rates for a month. Notably, if there is already an operating flare on site, or a combustor or other combustion device is used that does not require pilot and purge flows, then GHG emissions would always be reduced by burning the vent gas, no matter the duration of the venting event.

Table S1. Analysis of detected and quantified methane emissions from individual flares and vent stacks.

Facility ID	Description on Facility Approval	Field Observations	Mean Emission Rate [kg/h]	Duration between flights [h]	Venting Duration to Justify Month of Flaring [h][a]
Unlit Flares					
BC_358	Continuous flare	Unlit and detected in both flight and reflight	114.9	23.9	0.6
BC_247	Continuous flare	Unlit and detected in both flight and reflight	55.1	234.9	1.3
BC_367	Continuous flare	Unlit and detected in both flight and reflight[b]	50.1	73.3	1.4
BC_271	Continuous flare	Unlit in both flights; detected in initial flight only	27.6	70.8	1.3[g]
BC_504	Continuous flare	Unlit and detected in both flight and reflight	25.8	73.3	2.7
BC_364	Intermittent flare	Unlit and detected in both flight and reflight[c]	18.3	73.3	3.9
BC_86	Continuous flare	Unlit and detected in both flight and reflight	18.1	77.3	3.9
BC_40	Continuous flare	Unlit and detected in both flight and reflight	11.1	167.1	6.4
BC_8	Continuous flare	Unlit and detected in both flight and reflight	8.8	146.1	8.0
BC_242	Continuous flare	Unlit and detected in both flight and reflight[d]	8.7	74.5	8.1
BC_87	Continuous flare	Unlit in both flights; detected in reflight only[e]	2.5	72.6	17.5[h]
Unlit Vent Stacks					
BC_359	Emergency Vent Stack	Unlit and detected in both flight and reflight	27.9	23.7	2.5
BC_506	Vent stack	Unlit and detected in both flight and reflight	9.6	126.9	7.4
BC_20	Vent stack	Unlit and detected during flight; not reflown[f]	2.7	n/a	25.9
Partially Lit Flares					
BC_318	Continuous flare	Lit during flight, unlit and emitting during reflight	10.7	77.7	n/a
BC_16	Continuous flare	Unlit and emitting during flight, lit during reflight	9.8	256.5	n/a
BC_137	Continuous flare	Unlit and emitting during flight, lit during reflight	2.3	96.8	n/a
Lit Flares with Detected Methane Emissions					
BC_124	Pit Flare[i]	Lit and emitting during both flight and reflight	3.6	96.5	n/a
BC_94	Continuous flare	Lit and emitting during both flight and reflight	3.5	95.9	n/a

[a] Hours at measured venting rate beyond which net greenhouse gas emissions would be lower via flaring even if operating a 4-inch diameter flare continuously on standby for a month at recommended maximum pilot and purge flow rates.
[b] Ground team noted that this flare "is only used as a vent stack and is continuously venting".
[c] Ground team noted this flare is being used as a vent stack.
[d] This flare was surveyed in 2019 [11] and observed to be lit without detected emissions.
[e] This flare was surveyed in 2019 [11] and visibly unlit and emitting methane at 5.0 kg/h. However, ground team noted that the pilot flame was operating and visible in their subsequent visit after the flights in 2021.
[f] This vent stack was surveyed in 2019 [11] and not detected.
[g] Based on measured emission rate of 52.4 kg/h during initial flight.
[h] Based on measured emission rate of 4.0 kg/h during reflight.
[i] This is reportedly a legacy facility as pit flares have not been approved for several years and may only be constructed if "specifically authorized" [12].

S3 Total Site Tank Emissions

Figure S2 plots the distribution of aerial measured tank emissions by site. In this figure, the measured emission for all tanks at each site have been aggregated to permit comparison with upcoming regulated site limits for total tank emissions.

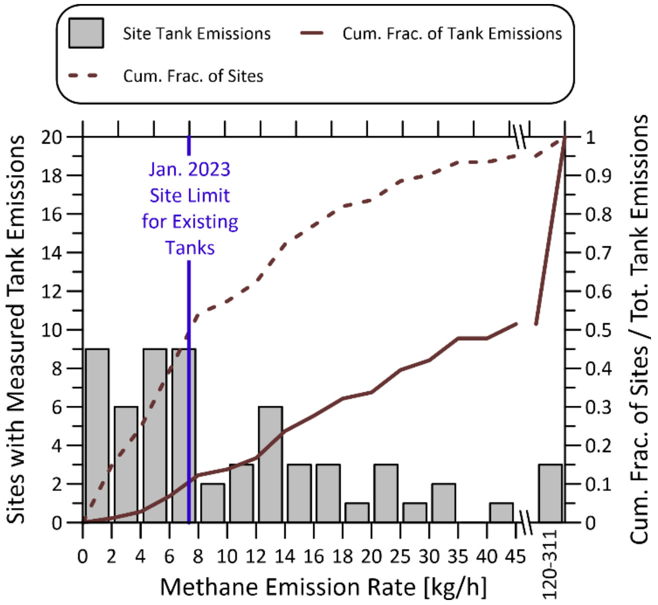

Figure S2. Distribution of total measured tank emissions at individual sites and overlaid with forthcoming regulated limit for existing tanks of 9000 m3/mo (whole gas), which is converted to a methane emission rate of 7.36 kg/h assuming a representative methane fraction of 88%.

As noted in the main text, half (52%) of sites in the survey had aggregate measured tank emissions above the upcoming limit of 9000 m^3/mo (whole gas, equivalent to 7.36 kg/h of methane), and these sites represent 90% of the total measured methane from tanks. In principle, bringing these sites into compliance with the limit could reduce emissions by 70%. Unfortunately, closer analysis of the data suggests this is not realistic given that approximately 65% of these excess tank emissions are from tanks that already have controls and are currently subject to regulated three times per year leak detection and repair (LDAR) surveys. Ideally, if regulations were working as intended, these emissions should already be reduced toward zero. Moreover, if the current measured population average for *controlled* tanks is taken as the achievable limit without additional MRV, then the upcoming tank limit is only likely to net a reduction of 27%.

S4 References

(1) Johnson, M. R.; Conrad, B. M.; Tyner, D. R. Creating Measurement-Based Oil and Gas Sector Methane Inventories Using Source-Resolved Aerial Surveys (Submitted). *Submitt. to Commun. Earth Environ. (preprint https//doi.org/10.21203/rs.3.rs-2203868/v1)* **2022**. https://doi.org/10.21203/rs.3.rs-2203868/v1.

(2) Forster, P.; Storelvmo, T.; Armour, K.; Collins, W.; Dufresne, J.-L.; Frame, D.; Lunt, D. J.; Mauritsen, T.; Palmer, M. D.; Watanabe, M.; Wild, M.; Zhang, H. Chapter 7: The Earth's Energy Budget, Climate Feedbacks, and Climate Sensitivity. In *Climate Change 2021: The Physical Science Basis. Contribution of Working Group I to the Sixth*

Assessment Report of the Intergovernmental Panel on Climate Change; Masson-Delmotte, V., Zhai, P., Pirani, A., Connors, S. L., Péan, C., Berger, S., Caud, N., Chen, Y., Goldfarb, L., Gomis, M. I., Huang, M., Leitzell, K., Lonnoy, E., Matthews, J. B. R., Maycock, T. K., Waterfield, T., Yelekçi, O., Yu, R., Zhou, B., Eds.; Cambridge University Press, 2021.

(3) CAPP. *Fuel Gas Best Management Practices: Module 4 - Efficient Use of Fuel Gas in Flaring Operations*; Canadian Association of Petroleum Producers (CAPP), 2008.

(4) Sorrels, J. L.; Coburn, J. Section 3 - VOC Controls; Section 3.2 - VOC Destruction Controls; Chapter 1 - Flares. In *EPA Air Pollution Control Cost Manual*; U.S. Environmental Protection Agency (US EPA), 2019; p 71.

(5) Shore, D. Making the Flare Safe. *J. Loss Prev. Process Ind.* **1996**, *9* (6), 363–381. https://doi.org/10.1016/S0950-4230(96)00026-5.

(6) AER. *Directive 060*; Alberta Energy Regulator (AER): Calgary, AB, 2021.

(7) Tyner, D. R.; Johnson, M. R. *Improving Upstream Oil and Gas Emissions Estimates with Updated Gas Composition Data*; Carleton University Energy & Emissions Research Lab.: Ottawa, ON, 2020.

(8) Burtt, D. C.; Corbin, D. J.; Armitage, J. R.; Crosland, B. M.; Jefferson, A. M.; Kopp, G. A.; Kostiuk, L. W.; Johnson, M. R. A Methodology for Quantifying Combustion Efficiencies and Species Emission Rates of Flares Subjected to Crosswind. *J. Energy Inst.* **2022**, *104*, 124–132. https://doi.org/10.1016/j.joei.2022.07.005.

(9) Johnson, M. R.; Wilson, D. J.; Kostiuk, L. W. A Fuel Stripping Mechanism for Wake-Stabilized Jet Diffusion Flames in Crossflow. *Combust. Sci. Technol.* **2001**, *169* (1), 155–174. https://doi.org/10.1080/00102200108907844.

(10) CSA. *Z620.3:22 Flaring, Incineration, and Enclosed Combustion*; CSA Group, 2022.

(11) Tyner, D. R.; Johnson, M. R. Where the Methane Is—Insights from Novel Airborne LiDAR Measurements Combined with Ground Survey Data. *Environ. Sci. Technol.* **2021**, *55* (14), 9773–9783. https://doi.org/10.1021/acs.est.1c01572.

(12) BCOGC. *Oil and Gas Activities Act Drilling and Production Regulation*; British Columbia Oil and Gas Commission: British Columbia, 2020.

S5 Underlying Data Tables

Table S2: Partial sample of supplemental aerial measurement data by source. Complete data are provided in a separate .xlsx file.

Anonymized Site ID	Anonymized Source ID	Anonymized Equipment ID	Emission Rate (Monte Carlo Average) [kg/h]	Emission Rate (2.5th Percentile from Monte Carlo) [kg/h]	Emission Rate (97.5th Percentile from Monte Carlo) [kg/h]	Source Description	Is 2021 Ground Site
BC_1	Src_1	A	11.7	7.9	17.7	Compressor Buildings	Yes
BC_1	Src_2	B	13.7	9.3	20.4	Dehydrators	Yes
BC_1	Src_3	A	2.8	1.6	5.0	Compressor Buildings	Yes
BC_1	Src_4	C	1.3	0.5	2.4	Piping	Yes
BC_1	Src_5	D	0.4	0.1	0.8	Power Generators	Yes
⋮	⋮	⋮	⋮	⋮	⋮	⋮	⋮
BC_502	Src_539	A	4.7	2.3	9.3	Compressor Buildings	No
BC_504	Src_540	A	25.8	12.0	52.4	Flares (Unlit/lit)	No
BC_504	Src_541	B	2.8	1.2	5.8	Separators	No
BC_506	Src_542	A	5.5	2.4	11.4	Tanks	Yes
BC_506	Src_543	B	9.6	4.6	18.7	Flares (Unlit/lit)	Yes

Table S3: Partial sample of supplemental aerial measurement data by pass. Complete data are provided in a separate .xlsx file.

Anonymized Site ID	Anonymized Source ID	Anonymized Equipment ID	Days Since Initial Site Measurement Day	Aircraft Altitude Above Ground Level [m]	Estimated Wind Speed[a] at 3 m [m/s]	Estimated Emission Rate[b,c,d] [kg/h]	Source Description	Is 2021 Ground Site
BC_1	Src_1	A	0	162.8	2.57	Not Detected	Compressor Buildings	Yes
BC_1	Src_1	A	0	173.1	2.61	8.18	Compressor Buildings	Yes
BC_1	Src_1	A	0	181.7	2.65	8.04	Compressor Buildings	Yes
BC_1	Src_1	A	0	166.6	2.67	9.30	Compressor Buildings	Yes
BC_1	Src_1	A	0	158.0	2.68	10.50	Compressor Buildings	Yes
⋮	⋮	⋮	⋮	⋮	⋮	⋮	⋮	⋮
BC_506	Src_542	A	0	155.1	4.68	16.97	Tanks	Yes
BC_506	Src_542	A	5	161.2	3.52	Not Detected	Tanks	Yes
BC_506	Src_543	B	0	154.3	4.65	1.37	Flares (Unlit/lit)	Yes
BC_506	Src_543	B	0	155.1	4.68	12.42	Flares (Unlit/lit)	Yes
BC_506	Src_543	B	5	161.2	3.52	13.98	Flares (Unlit/lit)	Yes

Table S4: Partial sample of supplemental compressor inventory data from ground follow-ups. Complete data are provided in a separate .xlsx file.

Anonymized Site ID	Ground Inspection Date	Compressor Make	Compressor Model	Engine Make	Engine Model	Engine Driver Type	Enging Power (nameplate) [bhp]	Estimated Full-Load Engine Emissions Derived from Available/Obtainable Manufacturer Specifications for Compressor Packages with Aerial-Detected Emissions[a] [kg/h]	Compressor Is Controlled	Compressor Emission Control Method	Has Fuel-Air Control Panel	Total Aerially Detected Emissions (MC-average) [kg/h]
BC_1	2021-09-30	Ariel	JGK/4	Waukesha	L7042GSI ESM	Natural Gas	1480	3.11	No		Yes	14.49
BC_2	2021-09-30	Ariel	JGK/4	Waukesha	L7042GSI ESM	Natural Gas	1658	b	Yes	Low Pressure Flare	Yes	0.00
BC_3	2021-09-30	Ariel	JGK/4	Waukesha	L5774LT	Natural Gas	1280	b	Yes	Low Pressure Flare	Yes	0.00
BC_4	2021-09-30	Ariel	JGK/4	Waukesha	L7042GL	Natural Gas	1480	6.66	Yes	Low Pressure Flare	Yes	9.16
BC_4	2021-09-30	Ariel	JGK/4-2	Waukesha	L5774LT ESM	Natural Gas	1280	b	Yes	Low Pressure Flare	Yes	0.00
⋮	⋮	⋮	⋮	⋮	⋮	⋮	⋮	⋮	⋮	⋮	⋮	⋮
BC_501	2021-09-23	Ariel	JGK/4	Waukesha	L7042GL	Natural Gas	1478	6.66	Yes	Low Pressure Flare	Yes	11.33
BC_501	2021-09-23	Ariel	JGK/4	Waukesha	L7042GL ESM	Natural Gas	1480	5.80	Yes	High Pressure Flare	Yes	13.04
BC_501	2021-09-23	Ariel	JGK/4	Waukesha	L7042GL ESM	Natural Gas	1480	5.80	Yes	Low Pressure Flare	Yes	12.13
BC_501	2021-09-23	Ariel	JGA/2	General Electric	5KS324STE205A	Electric			Yes	Low Pressure Flare	No	0.00
BC_506	2021-09-23	Ariel	JGK/4	Waukesha	L7042GSI	Natural Gas	1480	b	No		Yes	0.00

Table S5: Partial sample of supplemental tank inventory data from manual inspections of high-resolution aerial imagery and facility plot plans made available by BC OGC. Complete data are provided in a separate .xlsx file.

Anonymized Site ID	Controlled Production Tanks	Uncontrolled Production Tanks	Total Production Tanks	Controlled Dehydrator Tanks	Uncontrolled Dehydrator Tanks	Total Dehydrator Tanks	Controlled Amine Tanks	Uncontrolled Amine Tanks	Total Amine Tanks	Controlled Lube Oil Tanks	Uncontrolled Lube Oil Tanks	Total Lube Oil Tanks	Horizontal Storage Tanks	Other Tanks	Is 2021 Ground Site
BC_1	0	3	3	0	1	1	0	0	0	0	0	0	0	1	Yes
BC_2	1	0	1	0	0	0	0	0	0	0	0	0	0	0	Yes
BC_3	1	0	1	0	0	0	0	0	0	0	0	0	0	0	Yes
BC_4	2	0	2	0	0	0	0	0	0	0	1	1	0	0	Yes
BC_5	0	1	1	0	0	0	0	0	0	0	0	0	0	0	Yes
⋮	⋮	⋮	⋮	⋮	⋮	⋮	⋮	⋮	⋮	⋮	⋮	⋮	⋮	⋮	⋮
BC_504	0	2	2	0	0	0	0	0	0	0	1	1	0	1	No
BC_505	2	0	2	1	0	1	0	0	0	0	0	0	0	1	No
BC_506	0	1	1	0	1	1	0	0	0	0	0	0	0	1	Yes
BC_507	0	1	1	0	0	0	0	0	0	0	0	0	0	0	No
BC_508	13	0	13	0	0	0	0	0	0	0	0	0	0	8	No

Table S6: Partial sample of supplement pneumatic inventory data at separators and other sources (see Figure 5 in the main text) from ground follow-ups. Complete data are provided in separate .xlsx file.

Figure 5 Index	Anonymized Site ID	Aerial Measurement (MC-average) [kg/h]	Pnuematic Device	Make	Model	Supply Pressure [PSI]	Device Action	Multiple Actuations Observed over 15 Minutes	Estimated Emissions from Manufacturer Static Bleed Rates [kg/h]	Estimated Emissions Using Emission Factors from Field Studies [kg/h]	Footnote
1	BC_80	1.2	Not Inventoried	-	-	-	-	-	N/A	N/A	a
2	BC_80	1.3	Level Controller	Fisher	FSL2-XB	40	Snap	No	0.03	0.11	a
3	BC_448	1.9	Level Controller	Norriseal	3RF14NSLDF-BG	32	Snap	No	0.00	0.12	a
3	BC_448		Level Controller	Norriseal	3RF14NSLDF-BG	30	Snap	No	0.00	0.12	a
3	BC_448		Level Shutdown	Norriseal	1001A	28		-	0.00	0.12	a
3	BC_448		Pressure Controller	Fisher	C1	32	Direct	-	0.10	0.04	a,d
⋮	⋮	⋮	⋮	⋮	⋮	⋮	⋮	⋮	⋮	⋮	⋮
27	BC_22	4.9	Level Controller	Fisher	FSL2-XB	32	Snap	No	0.03	0.11	a
27	BC_22		Pressure Controller	Fisher	4150KR	31	Reverse	-	0.14	0.25	a,d
27	BC_22		Pressure Controller	Fisher	4150KR	32	Reverse	-	0.14	0.25	a,d
27	BC_22		Pump	Williams	WRA1115MNNSEBB	80		-	0.58	0.58	a,c
27	BC_22		Temperature Controller	Kimray	HT12	30		-	0.05	0.02	a,h
